# The Effects of Yin, Yang and Qi in the Skin on Pain

**DOI:** 10.3390/medicines3010005

**Published:** 2016-01-29

**Authors:** James David Adams

**Affiliations:** University of Southern California, School of Pharmacy, 1985 Zonal Avenue, Los Angeles, CA, 90089-9121, USA; jadams@usc.edu; Tel.: +1-323-442-1362; Fax: +1-323-442-1681

**Keywords:** pain, skin, topical, liniment, acupuncture, transient receptor potential cation channels

## Abstract

The most effective and safe treatment site for pain is in the skin. This chapter discusses the reasons to treat pain in the skin. Pain is sensed in the skin through transient receptor potential cation channels and other receptors. These receptors have endogenous agonists (yang) and antagonists (yin) that help the body control pain. Acupuncture works through modulation of these receptor activities (qi) in the skin; as do moxibustion and liniments. The treatment of pain in the skin has the potential to save many lives and improve pain therapy in most patients.

## 1. Introduction

The ancient Chinese concepts of yin, yang and qi have been explained in modern scientific terms [1]. Yin is cold, wet and diminishes blood flow. Yang is hot, dry and increases blood flow. Yin can also be represented by an antagonist that decreases pain and inflammation by inhibiting a receptor, such as a transient receptor potential cation channel (TRP). Yang can be represented by an agonist that decreases pain and inflammation by increasing the activity of a receptor, such as an opioid receptor. Qi is the flow of signal transduction mechanisms that occurs in cells because of the actions of agonists and antagonists. When qi flows well, these signal transduction mechanisms result in less pain and inflammation. A balance of yin and yang is necessary for qi to flow well. Signal transduction mechanisms may influence yin and yang by altering the transcription or activation of TRP or other receptors. There is a pain cycle that starts in the skin, travels to the brainstem and brain, and returns to the skin [2]. This pain cycle can magnify pain if left unchecked. The pain cycle is caused when yin and yang are not in balance and qi does not flow correctly in the skin.

The gate control theory of pain presents the pain cycle in terms of the brainstem rather than the skin [3]. In this theory, pain is sensed in the brain not the skin, and can be modified by non-painful sensations in the skin, such as rubbing the skin after a painful incident. This theory contends that inhibitory interneurons in the brainstem can suppress the transmission of pain signals into the brain. The gate control theory is useful, but does not take into account the many pain sensors in the skin and the fact that the signals from these pain sensors can be modified in the skin [2]. The fact that the skin is involved in pain sensation is obvious, since injection of a local anesthetic into the skin causes analgesia near the site of injection. The current paper proposes a modification of the gate control theory in which the skin is involved in sensing and modifying pain signals. The brainstem can modify pain signals. The brain is responsible for processing and modifying pain signals.

It is well known that ice packs decrease pain and swelling soon after an injury. Later, heat packs may be applied in order to decrease pain and swelling. How do ice and heat work? They work through TRPs that are sensitive to cold and heat. Initial TRP activation by cold or heat results in TRP deactivation that causes pain relief and decreases inflammation. TRPs are the most abundant pain receptors in the body. They are located in the plasma membranes of many cells of the body and are sensitive to heat, cold, pain, mechanical stimulation and other stimuli. They are abundant in the skin where they are located in the terminals of sensory afferent neurons [2]. TRP activation causes pain. TRPs are unusual receptors in that activation by an agonist may cause them to deactivate. This can lead to pain relief for much longer than expected. TRPs are channels that allow calcium and other positive ions to enter sensory neurons. Excessive calcium permeability can cause apoptosis of sensory terminals. This causes long term pain relief until nerve terminals can be regenerated [4]. TRPs are also involved in inflammation in the skin and other sites in the body [2].

## 2. TRP Diversity and Neuronal Populations

There are at least 28 different TRPs in the skin (Table 1), most of which are pain receptors [5]. In other words, these receptors are activated by painful stimuli and transmit these pain stimuli to the brain. There is no evidence of tolerance to the analgesia produced by inhibiting or deactivating TRPs. The body makes agonists and antagonists for these receptors which are derived from arachidonic acid and other fats. There are transient receptor potential cation channel vanilloid receptors, TRPV1–6, that can be inhibited by plant derived compounds such as capsaicin and some monoterpenoids [5]. Several TRPV receptor subtypes are activated by heat. As heat is applied, the receptors are initially activated, then deactivated resulting in channel closing and analgesia. TRPV receptors respond to endocannabinoids, endovanilloids, cannabinoids, hydroperoxyeicosatetraenoic acid (HPETE) and hydroxyeicosatetraenoic acid (HETE) [5]. Mechanical stimuli, such as pressure on the skin, can also activate some vanilloid channels such as TRPV4. There is one transient receptor potential cation channel ankyrin receptor, TRPA1, which can be inhibited by some monoterpenoids, other plant derived compounds, arachidonic acid and some prostaglandins. TRPA1 also responds to cold and mechanical stimuli. There are transient receptor potential cation channel canonical receptors, TRPC1–7, that are inhibited by hyperforin and other compounds. TRPC5 is activated by low temperature. TRPC1 and 6 are activated by mechanical stretch. Transient receptor potential cation channel melastatin receptors, TRPM1–8, are inhibited by monoterpenoids such as menthol and by fats such as sphingosine. TRPM1, 2, 3, 4 and 5 are activated by heat. TRPM8 is activated by cold. The transient receptor potential cation channel polycystin receptors, TRPP1–3, may be inhibited by diterpenes such as triptolide. TRPP1 and 2 are mechanosensitive channels. The transient receptor potential cation channel mucolipin receptors, TRPML1–3, respond to protons.

**Table 1:** Skin Pain Receptors.

Receptor	Agonist	Antagonist
TRPV1–6	Heat, monoterpenoids, endocannabinoids, endovanilloids, capsaicin	Dynorphins, adenosine, resolvins
TRPA1	Cold, prostaglandins	Monoterpenoids, resolvins
TRPC1–7	Diacylglycerol	-
TRPM1–8	Heat, cold, steroids, menthol	-
TRPP1–3	Mechanical stress, calcium	-
TRPML1–3	Protons	-
CB1–2	Cannabinoids, endocannabinoids	-
EP1–4	Prostaglandins	-
Lipoxin A4 receptor and others	Lipoxins	-
Resolvin D1 receptor and others	Resolvins	-
H1, H3, H4	Histamine	Antihistamines
5-HT1AR, 5-HT2AR, 5-HT3R	Serotonin	Methysergide
BLTR1-2	Leukotriene B4	-
MOR, KOR, DOR, NOP	Enkephalins, endorphins, dynorphins, nociceptin, fentanyl	-
B1-2	Bradykinins	-
P2 receptors	ATP	-
*N*-type calcium channels	-	Gabapentin, ziconotide
Voltage gated ion channels	Aconitine, delphinine	Local anesthetics
NMDA, AMPA, Kainate	Glutamate	Ketamine, ibogaine

TRPV—transient receptor potential cation channel vanilloid, TRPA—transient receptor potential cation channel ankyrin, TRPC—transient receptor potential cation channel canonical, TRPM—transient receptor potential cation channel melastatin, TRPP—transient receptor potential cation channel polycystin, TRPML—transient receptor potential cation channel mucolipin, CB—cannabinoid receptor, EP—prostaglandin receptor, H—histamine receptor, 5-HTR—serotonin receptor, BLTR—leukotriene receptor, MOR—mu opioid receptor, KOR—kappa opioid receptor, DOR—delta opioid receptor, NOP—nociception receptor, B—bradykinin receptor, P2—adenosine triphosphate (ATP) receptor, NMDA—N-methyl-D-aspartate receptor, AMPA—alpha-amino-3-hydroxy-5-methyl-4-isoxazolepropionic acid receptor.

TRPs are found in non-overlapping populations of nerve terminals in the skin [6]. Many of these receptors also are found in endothelial and dendritic cells and are involved in dermatitis. Since these receptors are found in non-overlapping neuron populations, this makes pain treatment complex. For instance, if a menthol liniment is applied to the skin, it may not inhibit all of the neuronal terminals in the area, and may not adequately relieve pain. It is important to use a liniment that inhibits as many skin TRPs as possible in order to get pain relief, which may involve multiple monoterpenoids [7,8,9]. 

## 3. Other Skin Receptors Involved in Pain

The skin contains a variety of receptors involved in pain [10]. TRP receptors are the most abundant and varied. There are cannabinoid receptors, CB1 and CB2, which are involved in inflammation and pain sensation in the skin [11]. Cannabinoids are also agonists for several TRPs and cause an initial activation of the receptors followed by deactivation. There are skin lipoxin receptors that cause pain [12]. There are skin resolvin receptors that decrease pain [12]. Resolvins also inhibit TRPA1, TRPV3 and TRPV4. There are opioid receptors in the skin [13]. Opioid peptides such as the enkephalins are also made in the skin [13]. Fentanyl patches are used to treat pain and stimulate opioid receptors by direct application to the skin. Other pain receptors in the skin include bradykinin receptors, histamine receptors, serotonin receptors, adenosine triphosphate (ATP) receptors, prostaglandin receptors, *N*-type calcium channels, voltage gated sodium channels and glutamate receptors [10]. Voltage gated sodium channels are inhibited by local anesthetics and result in pain inhibition in the skin [10]. All of these receptors are involved in causing and relieving pain. All of these receptors have endogenous agonists and antagonists that are involved in pain and analgesia. Pain occurs when the production of these endogenous compounds is out of balance and causes too much stimulation of pain receptors.

## 4. Interactions of Pain Receptor Responses

The activation of one pain receptor can potentiate the activity of other pain receptors in the skin. This interaction greatly increases pain, in other words increases the improper flow of qi in the skin. TRPC5 activity is potentiated by Gq coupled G-protein coupled receptor (GPCR) activation [2], such as by glutamate, serotonin, histamine, muscarine and angtiotensin II receptors. TRPM2 is potentiated by receptor activation that involves the formation of cyclic-adenosine diphosphate (ADP) ribose, such as by muscarinic receptors [2]. Bradykinin receptor activation potentiates TRPA1 activity [2]. TRPC channels are activated by phospholipase C [14]. Several receptors activate phospholipase C, such as muscarinic, histaminergic, serotonergic, glutamatergic and muscarinic. Interactions of these pain receptors alter qi in the skin such that pain is magnified.

## 5. Skin Cyclooxygenase-2

Cyclooxygense-2 (COX2) is the main source of prostaglandins. As mentioned before, prostaglandins cause pain by interacting with prostaglandin receptors and TRPs. They also potentiate TRPV1 activity by increasing protein kinase A and C activity [2]. COX2 activity increases in the skin in chronic pain conditions [15]. This induction of skin COX2 makes pain worse and ensures that pain will continue until COX2 activity decreases. It is not known how skin COX2 is induced in chronic pain. Nonsteroidal anti-inflammatory agents (NSAIDS) are used to inhibit both COX1 and COX2 in the treatment of pain. These oral or injected agents do not penetrate adequately into the skin to inhibit skin COX2 [2]. On the other hand, topical sesquiterpenes are known to penetrate the skin and may inhibit COX2 or decrease the expression of COX2 in the skin [7,8]. Topical preparations can be superior to oral or injected preparations for pain treatment.

## 6. Moxibustion and Skin Yin, Yang and Qi

Moxibustion involves heating the skin at specific acupuncture points to relieve pain [16]. Application of heat for several seconds causes the deactivation of some TRPs [5]. This diminishes pain by decreasing the activity of skin sensory neurons. The heat sensitive TRPs are discussed above. By using a yang influence, heat, moxibustion reestablishes the proper flow of qi that comes from TRPs in sensory neurons and decreases pain.

## 7. Acupuncture and Skin Yin, Yang and Qi

Acupuncture causes an initial activation of several different TRPs by causing an initial pain sensation and a mechanical stimulation. Several TRPs are activated by pain and mechanical stimuli as discussed above. The continued application of the acupuncture needle for more than several seconds, may cause these channels to deactivate and relieve pain. As discussed before, the application of too much yin (antagonist) or yang (agonist) to TRPs, causes them to shut down. This relieves pain by allowing qi (signal transduction) to flow correctly. It has been suggested that mast cell degranulation involves TRPV2 activation and that acupuncture may increase this process [17]. Mast cell degranulation is important in skin inflammation, irritation and pain. In addition, electroacupuncture was shown to decrease the expression of TRPV1 [13]. COX2 expression can also be diminished by acupuncture [18,19,20,21], which is important in the treatment of chronic pain conditions. Since acupuncture is applied to the skin, it inhibits the expression of skin COX2 [21]. It has been known for centuries that acupuncture is useful in the treatment of acute and chronic pain. TRP inhibition and decreased COX2 expression may be involved in this pain relief.

## 8. The Superiority Pain Treatment in the Skin

The most popular treatments for pain are oral medications such as NSAIDS and opioids. These agents are given in large doses and cause many toxic reactions. NSAIDS cause 100,000 ulcers in the US every year that result in 10,000 deaths. NSAIDS also cause kidney damage and clotting problems that lead to stroke and heart attack. COX1 is the major source of thromboxane. COX2 is the major source of prostaglandins. The prostaglandin, prostacyclin (PGI2) can inhibit clotting. It is possible that inhibition of COX2 in platelets leads to excess thromboxane, inadequate PGI2 and clotting. NSAIDS alter the balance of arachidonic acid metabolites, including prostaglandins and lipoxins, such that drug toxicity occurs. Opioids cause tolerance, addiction and toxicity that leads to 14,000 deaths in the US every year. Opioid medicines alter the balance of natural opioid proteins in the body, including enkephalins and dynorphins, preventing the ability of the body to stop pain.

The gate control theory of pain supports the use of oral medications to treat pain, since the theory contends that pain is sensed in the brain and can be modified in the brainstem. The gate control theory has led to the overuse of oral pain medications and the many deaths discussed above.

In contrast, acupuncture is a safe treatment. Various liniments are available to treat pain by topical application [7,8,9]. These liniments are applied where they are needed, work quickly, and are very effective and safe.

## 9. Endovanilloids, Endocannabinoids and Arachidonic Acid Metabolites

The body has the ability to stop pain through a number of mechanisms (Table 1). The opioid peptides are important, including endorphins, enkephalins and dynorphins. They are produced in the brain and skin, work at opioid receptors in the brain and skin and are effective [10]. Several other compounds are made in the skin to either cause or inhibit pain sensed in the skin.

Endovanilloids work through deactivation of vanilloid receptors, the TRPV channels [22]. Several endovanilloids are made from arachidonic acid such as 15-hydroperoxyeicosatetraenoic acid (15-HPETE) made by 15-lipoxygenase, 12-HPETE made by 12-lipoxygenase, anandamide, and *N*-arachidonoyldopamine. 5-Lipoxygenase makes 5-HPETE, and leukotriene B4 that are both endovanilloids [23,24]. Other endovanilloids are not made from arachidonic acid and include *N*-oleoyldopamine, palmitoylethanolamide and the resolvins.

Lipoxins are trihydroxy-eicosatetraenoic acids made from arachidonic acid by 15-lipoxygenase and cause pain. They bind to several receptors such as, lipoxin A_4_ receptor, ALX/formyl peptide receptor 2, and resolvin D1 receptor [10]. Lipoxins also may act through stimulation of TRPV1 to cause pain [12]. They are made in very small amounts, where they are needed and are cleared quickly. They are balanced by the resolvins that inhibit pain.

Resolvins are trihydroxy-eicosapentaenoic acids made from omega-3 fatty acids by 15-lipoxygenase and COX2 [25]. Resolvins interact with several receptors, at least one of which is responsible for relieving pain. These receptors are resolvin D1 receptor, resolvin E1 receptor, G protein coupled receptor 32, lipoxin A_4_ receptor, chemokine like receptor 1 (also called chemerin receptor 23, ChemR23) and leukotriene B4 receptor [10]. Resolvins inhibit TRPA1, TRPV3 and TRPV4 which also relieves pain. One of the consequences of COX2 inhibition is that resolvin synthesis is inhibited, which alters the ability of the body to decrease pain.

Endocannabinoids include anadamide (made by phospholipase D), *N*-arachidonoyldopamine and 2-arachidonoylglycerol and interact with CB1 and CB2. Both of these receptors are found on sensory neurons in the skin and are involved in pain relief induced by cannabinoids and endocannabinoids [26]. It should be pointed out that although the endocannabinoids appear to be agonists for TRPV1, plant derived cannabinoids also interact with TRPV2, TRPV3, TRPV4, TRPA1 and TRPM8. It has been proposed that TRPV1 activity can be modulated through CB1 or CB2 activation by endocannabinoids [26].

All of these yin and yang influences alter the flow of qi. When qi flows correctly, pain is relieved. When qi flows incorrectly, pain occurs and may be magnified. Pain treatment should reestablish the proper balance of yin and yang. When the body is in balance, the body can heal itself [27,28].

## 10. Conclusions

In general, the inhibition of COX enzymes decreases prostaglandin and resolvin synthesis and shifts the metabolism of arachidonic acid to the lipoxygenase pathways. The synthesis of lipoxins and hepoxilins, mediators of pain and inflammation, may increase. Hepoxilins are made by 12-lipoxygenase [29]. However, the synthesis of several endocannabinoids and endovanilloids may increase, which decreases pain and inflammation.

There are several endogenous compounds that that come from omega-3 fatty acids through docosahexaenoic acid such as protectins and maresins. Protectins are antiapoptotic and anti-inflammatory [30]. Protectins are made by 17-lipoxygnease. Maresins are anti-inflammatory, analgesic and are made by macrophage 12-lipoxygenase [31]. The effects of COX inhibition on these compounds is not known.

The consequences of systemic inhibition of COX2 in the body are only now being understood. When orally active COX2 inhibitors, such as valdecoxib, were first released, thousands of patients experienced angina, heart attacks and other clotting issues. This may be due to inhibition of platelet COX2 that throws off the balance of PGI_2_ and thromboxanes, leading to clotting. Valdecoxib was removed from the US market in April of 2005. Since that time, clinical trials and meta-analyses have shown that all orally active or injected COX2 or nonselective COX inhibitors cause strokes, heart attacks and other clotting issues. Since July of 2015, the FDA requires the labels of all NSAIDS to indicate the increased risk of excess clotting. It is not known if inhibition of skin COX2 increases the risk of clotting. However, acupuncture inhibits skin COX2 and has been shown to not alter blood clotting [32].

TRP inhibition provides analgesia. Many TRP receptors are involved in this analgesia. It is important to inhibit as many types of TRP receptors as possible to provide effective pain relief, such as by using several monoterpenoids at the same time. Acupuncture and moxibustion provide pain relief, at least in part, through inhibition of TRP receptors.

The skin is a complex organ and is the major pain sensing organ. Many receptors that sense pain are found in the skin and are involved in the pain cycle that can magnify pain. These receptors interact with each other through signal transduction pathways. These pathways can be used to decrease sensitivity to pain such as by acupuncture, the application of cold, heat or topical preparations. This approach to pain treatment is safer and can be more effective than the use of oral or injected drugs.
